# Sympatry leads to reduced body condition in chickadees that occasionally hybridize

**DOI:** 10.1002/ece3.8756

**Published:** 2022-04-01

**Authors:** Kathryn C. Grabenstein, Ken A. Otter, Theresa M. Burg, Scott A. Taylor

**Affiliations:** ^1^ 1877 Ecology and Evolutionary Biology University of Colorado at Boulder Boulder Colorado USA; ^2^ Natural Resources and Environmental Studies University of Northern British Columbia Prince George British Columbia Canada; ^3^ University of Lethbridge Lethbridge Alberta Canada

**Keywords:** body condition, hybridization, interspecific competition, range edges, skyward contractions, species distributions, sympatry

## Abstract

Both abiotic and biotic drivers influence species distributions. Abiotic drivers such as climate have received considerable attention, even though biotic drivers such as hybridization often interact with abiotic drivers. We sought to explore the (1) costs of co‐occurrence for ecologically similar species that hybridize and (2) associations between ecological factors and condition to understand how abiotic and biotic factors influence species distributions. For two closely related and ecologically similar songbirds, black‐capped and mountain chickadees, we characterized body condition, as a proxy for fitness, using a 1358‐individual range‐wide dataset. We compared body condition in sympatry and allopatry with several abiotic and biotic factors using species‐specific generalized linear mixed models. We generated genomic data for a subset of 217 individuals to determine the extent of hybridization‐driven admixture in our dataset. Within this data subset, we found that ~11% of the chickadees had hybrid ancestry, and all hybrid individuals had typical black‐capped chickadee plumage. In the full dataset, we found that birds of both species, independent of demographic and abiotic factors, had significantly lower body condition when occurring in sympatry than birds in allopatry. This could be driven by either the inclusion of cryptic, likely poor condition, hybrids in our full dataset, competitive interactions in sympatry, or range edge effects. We are currently unable to discriminate between these mechanisms. Our findings have implications for mountain chickadees in particular, which will encounter more black‐capped chickadees as black‐capped chickadee ranges shift upslope and could lead to local declines in mountain chickadee populations.

## Graphical abstract

Understanding how species are distributed across landscapes, the relative influence of abiotic and biotic factors, and the consequences of overlap, is a central goal of evolutionary ecology, especially as ranges shift in response to rapid global change. We compared body condition between black‐capped and mountain chickadees in sympatry and allopatry to several demographic factors of known importance, as well as to abiotic factors, and controlled for hybrid status in a subset of the data using genome‐spanning loci. We found that, for both species, birds in sympatry, regardless of sex or age, had significantly lower body condition than birds in allopatry, and that this pattern might be driven by either the inclusion of cryptic, likely poor condition, hybrids in our full dataset, competitive interactions in sympatry, or range edge effects.

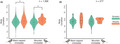

## 1 INTRODUCTION

Darwin (1859) originally emphasized the importance of abiotic factors for shaping species ranges. This idea has continued into modern‐day explorations of species distributions, which regularly involve assessing the influence of climate variables. This is particularly true for recent studies motivated by the pressing need to understand how a rapidly changing global climate will shift species ranges (Chen et al., 2011; Tingley et al., 2012). However, species rarely exist in isolation and empirical work has clearly demonstrated the ability of biotic interactions, independent of abiotic factors, to constrain species ranges (e.g., Benning et al., 2019; Blois et al., 2013; Harley, 2011; Pigot and Tobias, 2013). Nearly all populations co‐exist with other populations and species interactions have the potential to shape individual fitness, the evolutionary trajectories of populations, and, ultimately, species ranges (Stuart et al., 2014; Weber et al., 2017). Thus, considering the influence of both biotic and abiotic factors on population dynamics is a central component to understanding how species are distributed across landscapes (Louthan et al., 2015).

Hybridization between closely related species is a unique example of how the interplay between ecology and evolution shapes species ranges and is, perhaps unsurprisingly, sensitive to changing climate (Taylor et al., 2015; Taylor, White, et al., 2014). Hybridization occurs when closely related, often ecologically similar, species interbreed to produce offspring (Harrison, 1990). Importantly, hybridization often occurs at range edges in regions of low population density or natural environmental transitions (Swenson & Howard, 2005). These environmental transitions are highly sensitive to shifts in global climate (Brice et al., 2020) so as changes in abiotic drivers (such as temperature and precipitation) shift species distributions and modify range overlap between closely related species, we expect changes in interspecific interactions in these transition zones such as increasing hybridization (Taylor et al., 2015).

Meta‐analyses suggest that the density of the hybridizing species plays a role in the directionality, and likely the frequency, of hybridization: namely, females of the rarer species often mate with males of the more common species (Wirtz, 1999). Thus, hybridization along range edges might be density dependent. Alternatively, hybridization along range edges might be driven by edge‐effects mediated by individual fitness (e.g., body condition) since individuals at range edges are hypothesized to be in worse condition than individuals at range centers (Sexton et al., 2009). If body condition influences mating decisions, hybridization at range edges could be a result of reduced body condition if females forgo reproduction with conspecifics in poor condition to mate with high condition heterospecifics (Pfennig, 2007). Although challenging, discriminating the relative roles of population‐level traits (e.g., species abundance) versus individual traits (e.g., fitness, competitive ability) as mechanisms promoting hybridization at range edges, and how these mechanisms will respond to changing climate, is an area of active research.

The challenges of assessing the mechanisms driving hybridization are further exacerbated by logistical difficulties in measuring how fitness varies for closely related species across entire ranges. Documenting signals of reduced fitness when closely related species co‐occur, and might be competing, is necessary for understanding the influence of biotic factors on species ranges (Hargreaves et al., 2020). However, the laborious nature of field studies that compare fitness measures between ecologically similar species within and outside of range overlap means these are often restricted to small portions of each species range (Lee‐Yaw et al., 2016). Although fitness underpins nearly every component of ecology and evolutionary biology, it is often difficult to define, and equally challenging to measure, especially for wild animal populations. Given the constraints of measuring fitness in wild populations of long‐lived organisms, body condition (e.g., mass relative to frame size) is often used as a proxy for fitness (Stevenson & Woods, 2006). Body condition—the physiological state of an individual that reflects how successfully they interact with their environment (Milenkaya et al., 2013)—is considered a reliable indicator of fitness in cases where individual survival and lifetime reproductive success cannot be measured (i.e., single capture).

**FIGURE 1:**
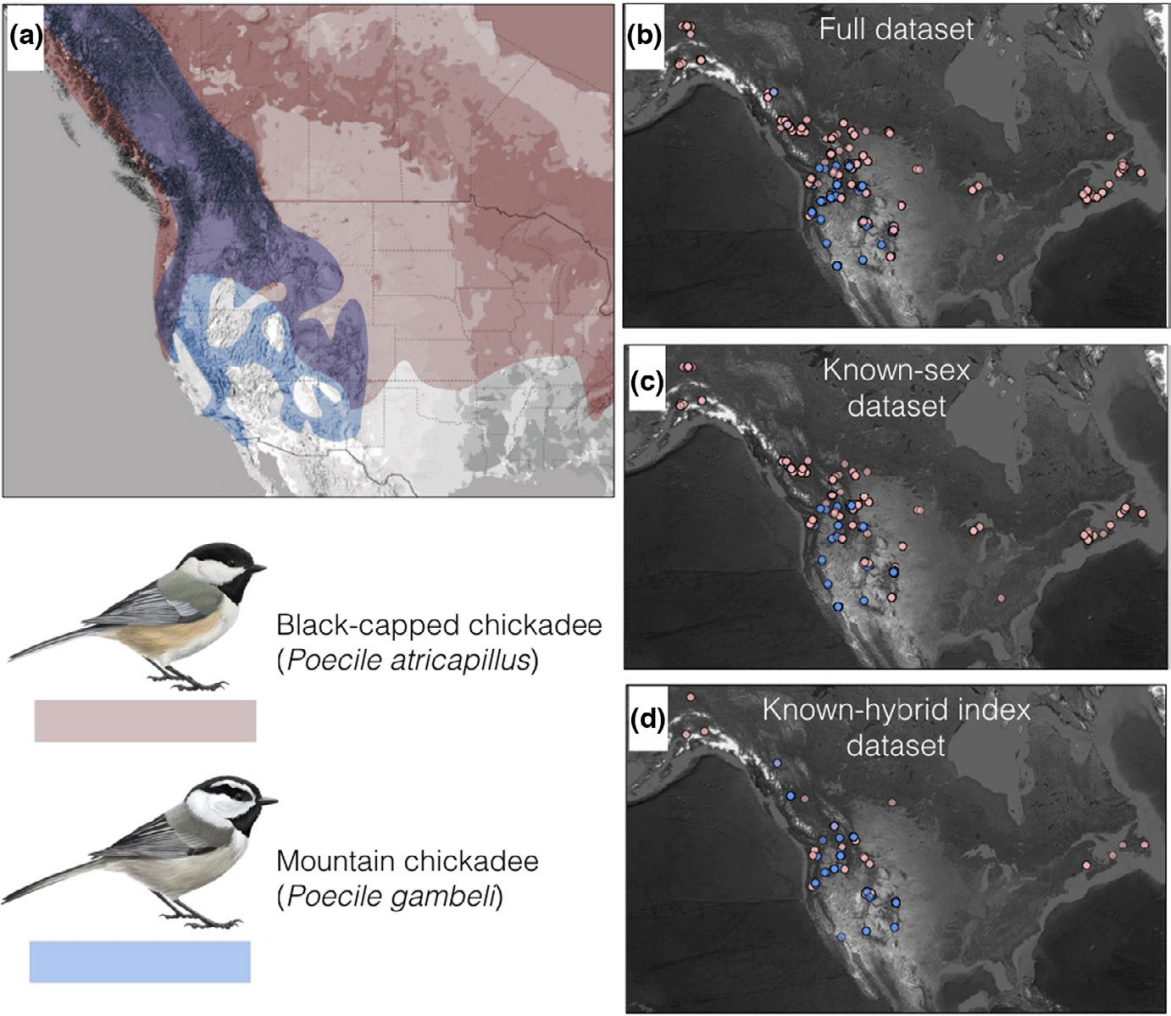
Maps of species ranges and sampling locations. (a) Black‐capped chickadee range in pink, mountain chickadee range in blue, and extensive range overlap in purple. These species are often segregated locally by elevation, but there is extensive overlap in transitional forest habitat. (b) Sampling locations for individuals included in the full dataset, (c) the known‐sex subset, and (d) the known‐hybrid index subset. Chickadee illustrations by Jessica French

Black‐capped (*Poecile atricapillus*) and mountain (*P. gambeli*) chickadees are closely related songbirds that share similar ecology. Both species are resident, social songbirds that occupy broad range distributions across western North America, with substantial areas of range overlap throughout nearly all the Rocky Mountains (Figure 1a). However, where their ranges overlap, the two species tend to occupy different habitats and are effectively separated along elevational gradients. Mountain chickadees typically occupy higher elevation conifer forests, with black‐capped chickadees occupying lower elevation mixed‐wood forests; sympatry occurs in mid‐elevation habitats where these two habitats converge. The width of the transitional habitat where both species co‐occur varies across their range overlap. In British Columbia, near the northern extent of the mountain chickadee distribution, the zone of overlap appears to be only a few hundred meters (pers. obs. K. Otter), whereas in southern parts of both species ranges in Colorado's Front Range the species co‐exist and breed throughout >1200 m of transitional habitat (pers. obs. K. Grabenstein).

Both chickadee species form stable, linear dominance hierarchies in nonbreeding flocks that solidify via competition for food resources and have been well‐characterized (Grava et al., 2013; Smith, 1976; Snell et al., 2016). Within single‐species flocks, older males are typically the most dominant individuals (Ratcliffe et al., 2007). Individuals of both sexes prefer dominant individuals and mate choice in both species is driven by dominance interactions within nonbreeding season flocks, with the most dominant female pairing with the most dominant male (Bonderud et al., 2018; Otter et al., 1998). How body condition influences dominance in chickadees remains less clear but may vary seasonally depending when mass is weighed (winter or spring); when comparing birds in the breeding season, such as in this study, dominant black‐capped chickadees do appear to be in better condition relative to subordinate males (Van Oort et al., 2007). Social dominance can also be compared across species; within mixed‐species groups, black‐capped chickadees are dominant over mountain chickadees, regardless of age or sex (Grava et al., 2012). This interspecific dominance relationship can also influence interspecific mating. Hybrid black‐capped/mountain chickadees documented with genetic data in a single study were always produced via copulations outside of the social pair, and found in mountain chickadee nests sired by black‐capped chickadee males (Grava et al., 2012). This suggests that hybrids are mainly produced through extra‐pair copulations, potentially because female mountain chickadees seek out copulations with more dominant male black‐capped chickadees. Thus, body condition might influence dominance rank through size differences, which in turn influence mating dynamics within this system.

Given that sympatry occurs along both species range edges and the biotic interactions within and between black‐capped and mountain chickadee flocks are well‐characterized (Dixon, 1965; Grava et al., 2012; Minockl, 1972; Ratcliffe et al., 2007), chickadees are well‐suited to explore the costs of co‐occurrence (i.e., whether individuals in mixed populations suffer reduced body condition). Furthermore, whether a reduction in condition for individuals occupying sympatric zones (which strongly correlates with transitional habitat at range edges) occurs for both species or only one species is unknown. Given that sympatry occurs along both species range edges, if suboptimal habitat, independent of competitive interactions, is driving individual condition, we would expect both species to suffer costs in sympatry and for that to correlate with suboptimal elevations (low‐elevation habitat for mountain chickadees and high‐elevation habitat for black‐capped chickadees). This expectation matches findings from previous studies of black‐capped chickadees where males breeding in young forests, which are considered poor quality habitat, were in worse condition than males breeding in mature forest, suggesting a cost to fitness for breeding in suboptimal habitat (Van Oort et al., 2007). Alternatively, if individual condition is instead influenced by competition between the two species, we would anticipate lower body condition for only the subordinate species (here, mountain chickadees) in sympatry, independent of elevation, which would potentially contribute to the patterns of asymmetrical hybridization observed in this system.

Using a 12‐year, range‐wide dataset of 1358 individuals, we characterized body condition, as a proxy for fitness, and compared it to demographic variables, as well as genomic data for a small subset of individuals, and abiotic factors, such as elevation, from across a substantial portion of the ranges of black‐capped and mountain chickadees (Figure 1). Ultimately, our investigation sheds insights into how both biotic (i.e., co‐occurrence with a closely related congener) and abiotic (i.e., elevation as a proxy for temperature and precipitation) factors shape species distributions in a rapidly changing world.

## 2 MATERIALS AND METHODS

### 2.1 Study system

While not sister taxa, black‐capped and mountain chickadees are closely related species (Harris et al., 2014) that share many morphological and behavioral traits (Grava et al., 2012; Hill & Lein, 1988; Lohr, 2008). Overall, body size and morphological differences are minimal with black‐capped chickadees weighing slightly less (11.37 ± 0.03 g vs. 11.59 ± 0.05 g) and having shorter tarsi (17.83 ± 0.05 mm vs. 18.62 ± 0.08 mm). Black‐capped chickadees are distributed continuously across the northern latitudes of North America while mountain chickadees occupy western mountain ranges. Both species have large range overlap in western North America and are often segregated along mountain slopes, with sympatry occurring within transitional habitat between the low‐elevation mixed forest occupied by black‐capped chickadees into the high‐elevation, xeric forests occupied by mountain chickadees.

Despite their broad range overlap, black‐capped and mountain chickadees appear to only hybridize occasionally (Howe, 1985; Hubbard, 1978; Martin & Martin, 1996), and more frequently in areas disturbed by human activities that occur within or near existing regions of range overlap (e.g., logging sites, urban centers, at transitional elevations; Grava et al., 2012). Investigations of continent‐wide patterns of hybridization in this system are limited (but see Graham et al., 2021), but human habitat disturbances might be promoting hybridization between the two species by altering the physical structure of the environment and driving competitive interactions in these altered habitats (Grabenstein & Taylor, 2018). This patchy hybridization is in stark contrast to the extensive hybridization that occurs between black‐capped and Carolina chickadees (*P. carolinensis*) along a narrow contact zone in eastern North America from Kansas to New Jersey (Reudink et al., 2007; Taylor et al., 2014). Genetic black‐capped/mountain chickadee hybrids documented in British Columbia were generated exclusively via extra‐pair copulations between male black‐capped chickadees and female mountain chickadees, but their plumage characteristics were unknown because they were sampled as chicks prior to complete plumage development (Grava et al., 2012). The only reported social pairing (as opposed to extra‐pair copulations) between these two species included a female mountain chickadee and a male black‐capped chickadee (Martin & Martin, 1996) along Colorado's Front Range and which matches expectations based on the previously described dominance hierarchy. The chicks from this pair had plumage very similar to black‐capped chickadees based on photos taken prior to fledging. Because black‐capped chickadees are socially dominant to mountain chickadees and females of both species use dominance status in mate choice, heterospecific male dominance interactions are hypothesized to drive hybridization in this system. However, the specific mechanisms driving this patchy pattern of hybridization remain largely unexplored.

### 2.2 Population sampling

As part of other ongoing projects in three research laboratories, we opportunistically sampled both black‐capped and mountain chickadees from across most of their contemporary North American distributions over a 12‐year period (2007–2016; 2018–2019) from May to August (i.e., during the breeding season) at 238 sites (*n* = 118 sympatric sites; *n* = 120 allopatric sites). Chickadees were identified to species using plumage characteristics in the field by trained individuals. The main sampling goal was to describe patterns of gene flow within both species’ ranges (Adams & Burg, 2015; Bonderud et al., 2018; Grava et al., 2012). Thus, our sampling did not focus exclusively on regions of overlap between the species and was relatively evenly distributed between species and sites through time, minimizing potential bias from only sampling in sympatry (Figures S1 and S2). Chickadees of both species were captured using audio lures at mist‐nets or baited Potter traps. Small blood samples (<20 µl) were collected from the brachial vein from captured chickadees and stored either as whole blood in 2% lysis buffer, ethanol, or blood on filter paper stored in ethanol. Tissue samples, pectoral muscle, were stored in ethanol. Individual sex was determined using sex‐specific characteristics during the breeding season (e.g., brood patches for females and cloacal protuberances for males; Desrochers, 1990). Individuals lacking either a brood patch or obvious cloacal protuberance were classified as unknown sex. Age was determined using plumage (Meigs et al., 1983) and breeding characteristics. Individuals were classified as: hatch year (HY), after hatch year (AHY), and after second year (ASY) in the field. These age classes were then combined as (1) HY and (2) AHY/ASY for statistical analyses based on previous work showing that dominance status in chickadees correlates strongly with age, where HY individuals are the most subordinate individuals (versus AHY/ASY birds; Smith, 1976). We measured the length of the right tarsus to the nearest 0.01 mm and mass to the nearest 0.5 g. Evidence from a single study in black‐capped chickadees suggests that weight can increase by as much as 1 g (~8% total body mass) over the course of the day as individuals forage, with individuals gaining ~0.5 g (~4% total body mass) from sunrise to midday (Graedel & Loveland, 1995). While we did not record exact time of capture for each bird, all birds were captured between 05:00 and 13:00 and our methods for weighing birds was only accurate to 0.5 g. Thus, while we have not controlled for specific time of capture in our downstream analyses, we feel that we have reasonably minimized variation by standardizing the range of capture times. Furthermore, our method for weighing birds cannot characterize the total variation needed to see effects of time of day (finer than 0.5‐g intervals). Over the 12 years, multiple banders (*n* = 10) collected these size measurements using standard techniques and bander was included as a crossed random effect in downstream statistical analyses to control for tarsus variation from bander alone.

Birds were recorded as occurring either in sympatry or allopatry using current distribution maps, eBird observations, and whether or not individuals of both species were sighted and/or captured at a single site (Sullivan et al., 2009). If individuals from both species were captured in a single location, we scored them as sympatric, regardless of distribution maps or eBird data. This allowed for allopatry to occur within the range of overlap (i.e., at high‐elevation sites where only mountain chickadees were sampled, or at low‐elevation sites where only black‐capped chickadees were sampled). We sampled 582 black‐capped chickadees from sympatry and 431 from allopatry and 294 mountain chickadees from sympatry and 51 from allopatry. All protocols were approved by the University of Colorado, Boulder IACUC panel (protocol 2683), the University of Northern British Columbia ACUC (protocols 2004‐07; A2008.0109.002; 2011.05; 2014.06 & 2017.01), and the University of Lethbridge (protocols 1028 and 1504) animal care committees and all methods in this study were performed in accordance with relevant guidelines, permits, and regulations.

### 2.3 Calculating body condition

We used body size measurements to calculate the scaled mass index (SMI) of black‐capped and mountain chickadees as outlined in Peig et al. (2009). Body condition measures should control for the correlation between length and mass. Peig et al. (2009) developed SMI for calculating body condition that accounts for the covariation between length and mass measurements when measuring body condition by standardizing body mass to a fixed value of a length measurement based upon a scaling relationship between mass and length. We calculated SMI for chickadees using the equation:

$${\hat{M}}_{i}=M_{i}{\left[\frac{L_{0}}{L_{i}}\right]}^{b_{\text{SMA}}}$$

where *M_i_* and *L_i_* represent individual mass and tarsus length measurements, respectively. *b*_SMA_ is the scaling component estimated from the standardized major axis (SMA) regression of the ln*M* on ln*L* and ${\hat{M}}_{i}$ is the predicted body mass for individual *i* with a length measurement (here, tarsus) standardized to *L*_0_, a species‐specific mean tarsus length. Importantly, comparisons of body condition between groups can only be made when SMI is calculated using the same scaling component. To account for slight size differences between the species (and potential differences in fat storage), we calculated SMI separately for each species. To allow us to compare the condition within species (i.e., sex‐specific differences), we calculated a separate scaling component (*b*_SMA_) for each species (black‐capped chickadees = 1.09, mountain chickadees = 1.12) and used species‐specific tarsus length means (black‐capped chickadees = 17.83 mm, mountain chickadees = 18.62 mm) for *L*_0_ to calculate the SMI. We used individual mass (g) as *M_i_* and individual tarsus length (mm) as *L_i_*.

### 2.4 Measuring hybridization in chickadees

#### 2.4.1 DNA extraction and quantification

Previous studies exploring hybridization between black‐capped and mountain chickadees have relied on intermediate plumage, or several microsatellite markers, to diagnose hybrids, which is likely insufficient to confidently identify all hybrids (e.g., late generation backcrosses, etc.). To examine hybridization between black‐capped and mountain chickadees, we used a genomic approach to generate hybrid indices (HI; a measure of genomic admixture) for a subset of 217 chickadees for which we also had measures of SMI, from across both species’ ranges (including areas of sympatry and allopatry), using reduced‐representation sequencing. We extracted DNA from either whole blood or pectoral tissue samples using a salt‐precipitation protocol (Miller et al., 1988). Specifically, 40 µl of the blood sample or ~2 g of tissue were added to 200 µl of homogenizing solution (0.4 M NaCl, 10 mM Tris–HCl pH 8.0, and 2 mM EDTA pH 8.0), 20 µl of 20% SDS, and 10 µl of Proteinase K (20 mg/ml). We vortexed samples and incubated at 56°C overnight. To breakdown cell components and draw off DNA‐associated proteins, we removed samples from the heat block, vortexed them, and added 150 µl of 6 M NaCl salt solution to each sample. We then vortexed samples for 30 s and centrifuged them for 30 min at 25,161 *g* to spin down cell components. After centrifuging, we decanted the supernatant into clean, labeled 1.5‐ml tubes and added 2 µl of Glycoblue™ (Thermo Fisher Scientific) to co‐precipitate and stain the DNA. To precipitate the DNA from the supernatant, we added 1000 µl of cold 100% ETOH and incubated the samples in −20°C for 15 min. After incubating the samples, we centrifuged them for 30 min at 25,161 *g* to spin down the precipitated DNA. We then decanted off the supernatant and added 1000 µl room temp 70% ETOH to wash the DNA and remove remaining salt. We repeated this wash step as needed until no visible salt remained around the DNA pellet. After washing the DNA, we air‐dried the pellets for 10 min. Lastly, we resuspended the DNA pellet in 100 µl of TE buffer (10 mM Tris, 1 mM EDTA at pH 8–9) and incubated at 37°C for 15 min. Samples were incubated at 4°C overnight to fully dissolve the DNA pellet. We quantified DNA concentrations using a Qubit 3.0 fluorometer (Invitrogen).

#### 2.4.2 Library preparation and genomic sequencing

To generate genomic sequence data, we used double‐digest restriction site‐associated DNA sequencing (ddRAD) following the protocol of Peterson et al. (2012) with modifications as described in Thrasher et al. (2018). Because ddRAD digests DNA with two restriction enzymes, it is a cost‐effective approach for generating genomic sequences for large studies of nonmodel organisms. For each sample, we digested ~500 ng of DNA with the restriction enzymes *SbfI* and *MspI* (New England BioLabs). We ligated P1 adapters to 5′ end of digested DNA with a *SbfI* compatible overhang and an inline barcode (5–7 bp long) to identify individual samples bioinformatically later in the analysis and P2 adaptors to the 3′ end of the digested DNA with a *MspI* compatible overhang. We pooled samples with unique P1 barcodes into 22 different indexing groups after digestion/ligation. To remove enzymes and small DNA fragments, we purified DNA in each index group using 1.53 Agencourt AMPure XP beads (Beckman Coulter). To ensure the same loci are recovered in all index groups, we size‐selected fragments between 400 and 700 bp using Blue Pippin (Sage Science). To add the full Illumina TruSeq primer sequences and unique indexing primers into each library, we performed a low cycle number PCR with Phusion High‐Fidelity DNA Polymerase (New England BioLabs) with the following thermocycling profile: 98°C for 30 s followed by 11 cycles at 98°C for 5 s, 60°C for 25 s, and 72°C for 10 s with a final extension at 72°C for 5 min. We visualized amplified products on a 1% agarose gel and performed a final 0.73 AMPure cleanup to eliminate DNA fragments smaller than 200 bp. We visualized libraries on a fragment Bioanalyzer (Agilent Technologies) to determine fragment size distribution. Finally, all 22 index groups were combined at equimolar ratios and sequenced on one Illumina NextSeq 500 lane (single‐end, 150 bp) at the Cornell University Biotechnology Resource Center.

#### 2.4.3 Quality control and filtering

To demultiplex chickadee samples, we used the process_radtags command in STACKS 2.41 (Catchen et al., 2013). After demultiplexing, we trimmed and filtered sequence reads using a custom script. Specifically, we removed Illumina adapters in the TruSeq3‐PE.fa file using TrimmomaticSE (Bolger et al., 2014). First, we searched for seed matches allowing maximally one mismatch. We then removed both leading and trailing low‐quality bases (Phred scores <20). Using a sliding window trimming approach, we scanned sequence reads from the 5′ end in 4‐bp windows and removed sequence reads when the average Phred quality score fell below 20. Finally, we dropped any reads shorter than 36 bp long. We used fastqc (Andrews, 2010) to calculate quality scores. After filtering, we aligned reads to a high‐quality black‐capped chickadee reference genome (Wagner et al., 2020) using bwa mem (Li, 2013) and a custom script to create sam files. We converted sam files to bam files using samtools (Li et al., 2009). Next, we used picard‐tools v.2.8.1 (Broad Institute, 2019) to mark duplicates and add/replace read groups. Lastly, we called variants based on a previously assembled black‐capped chickadee reference genome (Wagner et al., 2020) with bcftools (Narasimhan et al., 2016) and the mpileup command resulting in 517,699 unique loci. After calling variants, we filtered out single nucleotide polymorphisms (SNPs) with a Phred Score below 30, loci with a minor allele frequency less than 0.01% and 50% missingness, and loci with a maximum depth of 10× and a minimum depth of 1x. We retained 33,289 SNPs after filtering and we converted our variant call format (vcf) file to STRUCTURE format using PGD Spider version 2.1.1.5 (Lischer & Excoffier, 2012) for downstream analyses.

#### 2.4.4 Generating hybrid indices

To identify hybrid chickadees in our dataset, we calculated HI using the R package gghybrid (Bailey, 2018) based on the method of Buerkle (2005) for 217 chickadees. ggHybrid uses Bayesian Markov chain Monte Carlo to estimate what proportion of alleles originate from a predefined parental population. We assigned reference parental populations as allopatric black‐capped chickadees from the eastern US populations (HI = 0) and allopatric mountain chickadees from California, USA (HI = 1). These reference parental populations were used to calculate HI for 217 chickadees for which we had genomic data but were not included in any other downstream analyses since we did not have the necessary demographic information or morphometric measurements needed to calculate SMI. To ensure we were only using informative alleles to calculate HI, we used VCFtools (Danecek et al., 2011) to calculate the fixation index (*F*_st_), a measure of population differentiation, per SNP. *F*_st_ ranges from 0 to 1, and values closer to 1 indicate fixed genomic differences between populations, or species in this case. After calculating *F*_st_ for each SNP, we filtered our VCF table by loci with *F*_st_ > 0.65 (*n* = 955) to improve our estimation of HI by only including highly differentiated loci. We did not use fixed loci (e.g., *F*_st_ = 1) for estimating HI because our reduced‐representation approach did not capture enough fixed alleles to inform HI (hybrid index) estimation. Finally, we used the esth function with a burn‐in of 3000 iterations and 6000 total iterations. We also followed the above approach of estimating HI using loci with *F*_st_ > 0.80 (*n* = 443), which yielded similar results to using loci with *F*_st_ > 0.65, but had larger confidence intervals, so we used HIs generated from loci with *F*_st_ > 0.65 for the final calculation. After generating HI, we rescaled hybrid index from 0 to 0.5 using the equation, *g*(*x*) = 0.5 − abs(*x* − 0.5) to facilitate downstream analyses (0 = pure parental population of either species and 0.5 = F1 hybrid). We considered any individual with HI ≥ 0.20 to be a hybrid, which conservatively captures the variation in hybrid status by including a range of hybrid classes (e.g., F1s, backcrosses; Burke & Arnold, 2003). Our cut‐off of HI ≥ 0.20 for hybrid classification is conservative since many second‐generation hybrids in a similar chickadee system have lower admixture proportions (McQuillan et al., 2017). Thus, an HI ≥ 0.20 reliably captures first‐generation hybrids, but likely excludes later generation hybrids. Given the low‐resolution of the ddRAD dataset that we are working with, we think being conservative in this respect is warranted.

### 2.5 Statistical analyses

For these analyses, we assumed that biotic interactions would occur in areas of sympatry. We constructed generalized linear mixed models and used an AIC model averaging approach to explore the demographic, genomic, and ecological factors associated with the observed patterns of reduced body condition (e.g., lower SMI) exhibited by both species in sympatry versus allopatry for three separate datasets: known‐sex dataset, full dataset, and known‐hybrid index dataset (total of six rounds of model selection: three datasets, then subset for each of the two species).

Before building models, we scaled all numerical fixed effects (i.e., elevation, latitude, and hybrid index) to have a mean of zero and a standard deviation of one (*Z*‐score) to improve model stability and to generate standardized βs that are directly comparable within a model (Schielzeth, 2010). We also removed any individuals with missing data, to make different models comparable. For each round of model averaging, we checked each global model for overdispersion and independence of fixed effects, examined residuals for appropriate fit, and confirmed other necessary assumptions. As we did not have *a priori* information about the relative effects of age, sex, allopatry, or hybrid status on body condition, we used an all‐subset candidate approach to generate a complete list of candidate models for each response variable (Lukacs et al., 2010). From this candidate list, we used a delta AICc cutoff of four to generate a top model set (Burnham et al., 2011). We then used a model averaging technique to generate parameter and error estimates for each term (Symonds & Moussalli, 2011). This method is considered more conservative than traditional model selection, or reliance on AIC alone to choose a single top model, particularly for complex, observational datasets with interactions (Lukacs et al., 2010; Nguefack‐Tsague et al., 2016; Symonds & Moussalli, 2011). Variables were considered strongly supported if they were included in the final list of full model‐averaged coefficients and if the confidence interval for the model‐averaged parameter estimate did not span zero. These full model‐averaged coefficients (i.e., more conservative than the conditional average) are reported in the main text (Galipaud et al., 2017).

The transition of black‐capped chickadee habitat into mountain chickadee habitat at the local scale occurs along elevational gradients as wetter, deciduous forest occupied by black‐capped chickadees transitions to xeric, coniferous forest occupied by mountain chickadees. Given these environmental transitions, we compared chickadee condition for both species using a separate model for each species along elevation (m), while controlling for latitude (degree) (high elevation at high latitudes are harsher habitats than high elevation at low latitudes), as a proxy for measuring chickadee condition along range edges. For each species, we ran a linear regression of chickadee body condition against elevation by latitude using the lmList function in the R package lme4 (Bates et al., 2012). We found a significant effect of elevation by latitude on chickadee condition for both species using the full dataset (black‐capped chickadees: β = −6.873e−05; *t*(1009) = −5.5, *p* = 3.89e−08; mountain chickadees: β = 7.971e−05; *t*(341) = 3.817, *p *= .00021) and subsequently included elevation*latitude as a main effect in both species’ downstream models.

### 2.6 Subset known‐sex individuals

To describe chickadee condition relative to demographic and ecological factors, we used separate generalized linear mixed models for each species to examine the association between SMI and (1) *sex* (2) *age*, (3) *sympatry*, and (4) elevation*latitude for a subset of individuals with known sex (265 sympatric and 228 allopatric black‐capped chickadees; 130 sympatric and 26 allopatric mountain chickadees). For each species, we used the package lme4 (Bates et al., 2012) in R v.3.5.1 (R Development Core Team, 2017) to fit a global generalized linear mixed model to assess how the SMI of individuals (continuous with normal distribution) varied as a function of the covariates: *sex* (categorical with two levels), *age* (categorical with two levels), *sympatry* (categorical with two levels), and elevation*latitude (continuous with normal distribution). To account for dependency of individuals captured in the same year, as well as the dependency of repeat captures of the same individuals, we included *year* as a crossed random effect (i.e., random intercept). For the mountain chickadee model, we included *band number* as a crossed random effect to account for variation described by repeat capture individuals. There were no repeat captures in the known‐sex black‐capped chickadee subset, so we did not include *band number* as a random effect. We did not include *site* as a random effect in either model because the fixed term elevation*latitude accounts for the same variation in sampling locality. To account for random variation from different banders collecting bird size measurements, we included *bander* as a final crossed random effect for both models. For both black‐capped and mountain chickadee‐specific models, we retained *year* as the only random effect in our best supported models since neither *bander* nor *band number* (for mountain chickadees) explained a significant amount of variation for either species.

### 2.7 Full dataset individuals

For both species, the fixed effect *sex* did not explain a significant amount of variation for the best performing models in the known‐sex subset, so we next expanded our dataset to include all sampled individuals (582 sympatric and 431 allopatric black‐capped chickadees; 294 sympatric and 51 allopatric mountain chickadees) and conducted the same approach as detailed above. Specifically, we constructed species‐specific global models for each species to explore how the SMI of individuals varied as a function of *age*, *sympatry*, and elevation*latitude. For both species’ fully fit models, we included *year*, *band number*, and *bander* as crossed random effects.

### 2.8 Subset known‐hybrid index individuals

Finally, we explored the influence of hybrid index on body condition in conjunction with demographic and ecological factors using 217 individuals for which we had both a measure of body condition and a genotype (55 sympatric and 14 allopatric black‐capped chickadees; 132 sympatric and 16 allopatric mountain chickadees). As above, we constructed species‐specific generalized linear mixed models to explore how SMI varied as a function of the covariates: *age*, *sympatry*, *hybrid index*, and elevation*latitude. For both species’ fully fit models, we included *year*, *band number*, and *bander* as crossed random effects.

## 3 RESULTS

### 3.1 Hybridization between black‐capped and mountain chickadees

We found evidence of hybridization between black‐capped and mountain chickadees sporadically throughout their ranges. Using 955 highly differentiated loci (*F*_st_ > 0.65), we calculated HI for 217 chickadees. After removing allopatric birds, since they do not have the opportunity to hybridize, we found that 11.8% of sampled black‐capped and mountain chickadees (20/170 sympatric individuals) had intermediate HI (HI ≥ 0.20). After scaling hybrid index from 0 to 0.5, hybrid index ranged from 0.0 to 0.43 for black‐capped chickadees and from 0.0 to 0.13 for mountain chickadees. This suggests that either hybrids backcross more frequently to black‐capped chickadees (which would match dominance predictions), or that hybrids that backcross to black‐capped chickadees are more likely to produce viable and fertile offspring than hybrids that backcross to mountain chickadees. All hybrids (HI ≥ 0.20) were assigned as phenotypic black‐capped chickadees in the field.

### 3.2 Range‐wide chickadee body condition

**FIGURE 2:**
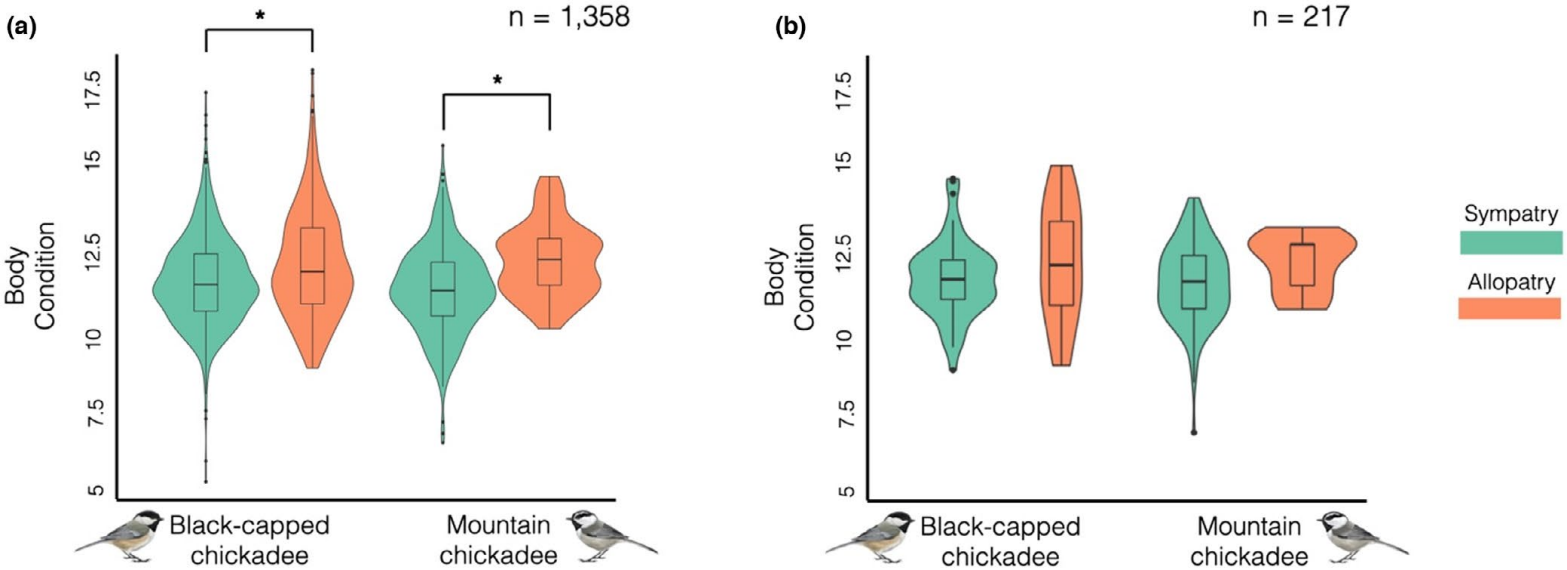
Individuals in sympatry had significantly lower body condition compared to birds in allopatry for both species (a) Black‐capped and mountain chickadees in sympatry were in worse condition than individuals in allopatry. We found sympatric black‐capped chickadees had on average 4.2% lower SMI scores than allopatric individuals (sympatric black‐capped chickadee SMI = 11.26 ± 0.05; allopatric black‐capped chickadee SMI = 11.73 ± 0.07; *t*(854) = −5.4735, *p* << .001). We also found that sympatric mountain chickadees had on average 7.7% lower SMI compared to mountain chickadees in allopatry (sympatric mountain chickadee SMI = 11.00 ± 0.07; allopatric mountain chickadee SMI = 11.85 ± 0.14; *t*(83) = −5.3649, *p* << .001). Violin plot of body condition (SMI, raw data) for black‐capped (left) and mountain chickadees (right) in sympatry (green) and allopatry (orange) from the full dataset (*n* = 1358). (b) For birds with known HI, no relationship between chickadee condition and any factors of biological interest. Violin plot of body condition (SMI, raw data) for black‐capped (left) and mountain chickadees (right) in sympatry (green) and allopatry (orange) for individuals with both known genotype and measured SMI (*n* = 217). Box plots denote means and 1st and 3rd quantiles overlaid. Chickadee illustrations by Jessica French

We found that when black‐capped and mountain chickadees co‐occur, all individuals are in significantly worse condition than when either species occurs alone, however, the factors associated with this pattern remain less clear. We calculated SMI for 1358 chickadees from 238 sites across North America (Figure 1b). We calculated SMI separately for each species to account for size differences between species using a separate scaling component ($b_{\text{SMA}}$) for each species ($b_{\text{SMA}}$ for black‐capped chickadees = 1.09, $b_{\text{SMA}}$ for mountain chickadees = 1.12) and species‐specific tarsus length means (black‐capped chickadee = 17.83 ± 0.05 mm; mountain chickadee = 18.62 ± 0.08 mm). We found a significant difference in SMI between allopatric and sympatric black‐capped chickadees, with sympatric black‐capped chickadees having on average 4.2% lower SMI scores than allopatric individuals (sympatric black‐capped chickadee SMI = 11.26 ± 0.05; allopatric black‐capped chickadee SMI = 11.73 ± 0.07; *t*(854) = −5.4735, *p* << .001; Figure 2). We also found that sympatric mountain chickadees had on average 7.7% lower SMIs compared to mountain chickadees in allopatry (sympatric mountain chickadee SMI = 11.00 ± 0.07; allopatric mountain chickadee SMI = 11.85 ± 0.14; *t*(83) = −5.3649, *p*  << .001; Figure 2).

### 3.3 Subset known‐sex individuals

#### 3.3.1 Black‐capped chickadees

Using only black‐capped chickadees with known sexes (*n* = 493), we found that body condition was lower for birds sampled in sympatry (sympatry: β = 0.38, SE = 0.14, *p* = .007, CI = 0.11, 0.66) and at higher elevations (elevation: β = −0.27, SE = 0.08, *p* = .005, CI = −0.41, −0.11; Table 1). However, we found no association between a bird's body condition and their age, sex, or sampling latitude.

**TABLE 1:** Model averaging results for the association between black‐capped chickadee scaled mass index and demographic and ecological factors using the known‐sex dataset

Term	Coefficient	Unconditional SE	Probability	Confidence interval
**Sympatry**	**0.38**	**0.14**	**0.007** ^*^	**0.11, 0.66**
**Elevation**	**−0.27**	**0.08**	**0.005** ^*^	**−0.41, −0.11**
Age	0.2005	0.169	0.236	−0.13, 0.53

Note

Strongly supported variables are highlighted in bold.

* Indicates significant model term.

#### 3.3.2 Mountain chickadees

Using only mountain chickadees with known sexes (*n* = 156), we found that older mountain chickadees had higher body condition than younger mountain chickadees (age: β = 1.12, SE = 0.50, *p* = .024, CI = 0.15, 2.10; Table 2). However, we found no association between a bird's body condition and their sex, whether they were sampled in sympatry, or their sampling location (elevation or latitude).

**TABLE 2:** Model averaging results for the association between mountain chickadee scaled mass index and demographic and ecological factors using the known‐sex dataset

Term	Coefficient	Unconditional SE	Probability	Confidence interval
**Age**	**1.12**	**0.50**	**0.024** ^*^	**0.15, 2.10**
Sympatry	0.55	0.31	0.08	−0.08, 1.12
Sex	−0.22	0.171	0.20	−0.55, 0.12

Note

Strongly supported variables are highlighted in bold.

* Indicates significant model term.

### 3.4 Full dataset individuals

#### 3.4.1 Black‐capped chickadees

Using all black‐capped chickadees in the full dataset (*n* = 1013), we found that the body condition of black‐capped chickadees sampled in sympatry was lower than individuals measured in allopatry (sympatry: β = 0.34, SE = 0.10, *p* = .00053, CI = 0.15, 0.53) and at higher elevations (elevation: β = −0.18, SE = 0.050, *p* < .0001, CI = −0.27, −0.091; Table 3). However, we found no association between a bird's body condition and their age or their sampling latitude.

**TABLE 3:** Model averaging results for the association between black‐capped chickadee scaled mass index and demographic and ecological factors using the full dataset

Term	Coefficient	Unconditional SE	Probability	Confidence interval
**Sympatry**	**0.34**	**0.10**	**0.00053** ^*^	**0.15, 0.53**
**Elevation**	**−0.18**	**0.050**	**<0.0001** ^*^	**−0.27, −0.091**

Note

Strongly supported variables are highlighted in bold.

* Indicates significant model term.

#### 3.4.2 Mountain chickadees

Using all mountain chickadees in the full dataset (*n* = 345), we found that body condition of mountain chickadees sampled in sympatry was lower than birds sampled in allopatry (sympatry: β = 0.51, SE = 0.24, *p* = .035, CI = 0.21, 0.94; Table 4). However, we found no association between a bird's body condition and their age or their sampling location (elevation or latitude).

**TABLE 4:** Model averaging results for the association between mountain chickadee scaled mass index and demographic and ecological factors using the full dataset

Term	Coefficient	Unconditional SE	Probability	Confidence interval
**Sympatry**	**0.51**	**0.24**	**0.035** ^*^	**0.21, 0.94**
Age	0.10	0.19	0.61	−0.094, 0.78
Elevation	−0.37	0.90	0.68	−3.72, 1.27
Latitude	0.00098	0.007	0.90	−0.031, 0.039
Elevation*Latitude	0.0083	0.021	0.69	0.031, 0.084

Note

Strongly supported variables are highlighted in bold.

* Indicates significant model term.

### 3.5 Subset known‐hybrid index individuals

#### 3.5.1 Black‐capped chickadees

Using only black‐capped chickadees with known HI and body condition scores (*n* = 101), we found no significant relationship between a bird's body condition and their age, whether they were sampled in sympatry, their hybrid index, or their sampling location (elevation or latitude; Table 5). However, our dataset is likely too small to detect an effect of hybrid index on condition given the relatively small number of hybrids we detected (*n* = 20).

**TABLE 5:** Model averaging results for the association between black‐capped chickadee scaled mass index and demographic and ecological factors using the known hybrid index dataset

Term	Coefficient	Unconditional SE	Probability	Confidence interval
Hybrid index	−0.46	1.086	0.68	−3.41, 1.85
Age	−0.066	0.19	0.73	−0.89, 0.32
Sympatry	−0.05	0.19	0.78	−0.95, 0.46
Elevation	−0.016	0.067	0.81	−0.44, 0.14

#### 3.5.2 Mountain chickadees

Using only known mountain chickadees with known HI and body condition scores (*n* = 116), we found no significant relationship between a bird's body condition and their age, whether they were sampled in sympatry, their hybrid index, or their sampling location (elevation or latitude; Table 6). As above, our dataset is too small to detect an effect of hybrid index on condition as we found no hybrids with mountain chickadee phenotypes.

**TABLE 6:** Model averaging results for the association between mountain chickadee scaled mass index and demographic and ecological factors using the known hybrid index dataset

Term	Coefficient	Unconditional SE	Probability	Confidence interval
Sympatry	−0.074	0.30	0.81	−1.26, 0.72
Age	−0.099	0.28	0.73	−1.19, 0.55
Elevation	0.049	0.11	0.65	−0.061, 0.47

## 4 DISCUSSION

In this study, we characterized body condition, as a proxy for fitness, of two closely related and ecologically similar songbird species that occasionally hybridize. Our dataset of 1358 individuals, which spans most of the breeding range of both species, collected over 12 years, provides a unique opportunity to explore body condition across a wide area of range overlap for a pair of birds that sporadically interbreed. By combining data on body condition with demographic and genomic data, we present evidence that black‐capped and mountain chickadees, identified to species using plumage, are in poorer condition when they occur in sympatry. The overall pattern of reduced body condition for both species in sympatry might be driven by (1) the inclusion of cryptic hybrids, which are presumably in poor condition, (2) competitive interactions between the species in sympatry, or (3) range edge effects; however, we cannot currently discriminate between these mechanisms. Hybrids occur at a higher frequency (11.8%) than expected in our smaller genotyped dataset, but the 217‐individual dataset is too small to determine whether the inclusion of hybrids in the larger dataset is driving the pattern (i.e., the broader pattern of lower condition in sympatry is not present in the 217‐individual dataset). Here, we discuss (1) how the inclusion of cryptic hybrids might drive the main pattern of reduced body condition in sympatry for all individuals, (2) how our results might be the effect of competitive interactions and/or (3) range edge effects, and (4) how our results may provide insight into impacts on populations in the context of rapid climate change and species range shifts.

We found that for both the known‐sex and full datasets for both species, black‐capped and mountain chickadees were in lower condition when they co‐occur than in sympatry (Table 7). Black‐capped chickadees in sympatry had on average 4.2% lower body condition scores than individuals in allopatry, which translates to being roughly 0.5 g lighter, assuming a species‐average tarsus length. Similarly, we found mountain chickadees in sympatry had on average 7.7% lower body condition scores, which is roughly 0.86 g lighter than birds in allopatry, assuming an average tarsus length for the species. The differences for both species’ body condition is less than the weight a bird gains throughout the day (~1 g increase during the day), but it is unclear how weight influences fitness in this system. When sampled during the breeding season, more dominant black‐capped chickadees are heavier for their size (i.e., in better condition) because they are not accumulating furcular fat to support overnight temperature maintenance during winter temperatures. Furthermore, weight likely impacts survivorship, with heavier birds being more likely to survive (Brittingham & Temple, 1988). Thus, birds in sympatry might have different survival rates compared to birds in allopatry due to differences in weight, but it is unclear how these differences in body condition translate to realized fitness differences between birds in sympatry and allopatry.

**TABLE 7:** Summary of six rounds of model averaging for each unique dataset and species combination

Dataset	Species	Sample size	Best model random terms	Model average fixed terms
Known‐Sex dataset	Black‐capped chickadee	493 total; Sympatric: 265 Allopatric: 228	Year	Sympatry Elevation
Known‐Sex dataset	Mountain chickadee	156 total; Sympatric: 130 Allopatric: 26	Year	Age Sympatry
Full dataset	Black‐capped chickadee	1013 total; Sympatric: 582 Allopatric: 431	Year	Sympatry Elevation
Full dataset	Mountain chickadee	345 total; Sympatric: 294 Allopatric: 51	Year	Sympatry
Known Hybrid Index dataset	Black‐capped chickadee	HI ≤ 0.20: 71 HI > 0.20: 20 Sympatric: 86 Allopatric: 15	Year	None
Known Hybrid Index dataset	Mountain chickadee	HI ≤ 0.20: 116 HI > 0.20: 0 Sympatric: 110 Allopatric: 6	Year	None

Black‐capped and mountain chickadees hybridize sporadically across areas of range overlap: ~11% of individuals sampled had HI ≥ 0.20 (Figure 1). Hybrid individuals from various taxonomic groups, including birds, are often in worse condition than either parental phenotype (Price & Bouvier, 2002). Unfortunately, due to small sample size (*n* = 217 individuals with both genomic and morphometric data) and weak power because we only detected 20 hybrid individuals, we could not determine whether the inclusion of cryptic hybrids in our full dataset explains the broader pattern of reduced body condition that we documented. Despite this, the documented pattern of reduced body condition for both black‐capped and mountain chickadees in sympatry could be driven by cryptic hybrids produced in sympatry that were classified as either parental species based on plumage.

Based on our HI cutoff of 0.2, all of the hybrid individuals we documented were classified by their morphology as black‐capped chickadees by trained researchers. That said, a less conservative cutoff of 0.1 would categorize several individuals identified as mountain chickadees by plumage as hybrids. Overall, the range of HI was greater for black‐capped (0.0–0.43) than mountain chickadees (0.0–0.13). Despite the widespread use of morphology for classifying hybrid black‐capped/mountain chickadees by the public, it is clear that morphology alone is not a reliable indicator of hybrid status for this species pair. Previous work has documented that hybrid offspring found in one population at a single site were sired exclusively by black‐capped chickadee males with mountain chickadee females through copulations outside of the social pair (Grava et al., 2012). The fact that all hybrid individuals (based on our 0.2 cutoff) exhibited black‐capped chickadee morphological characters suggest either differential survival of certain types of hybrids or that black‐capped chickadee plumage is dominant to mountain chickadee plumage. The wider distribution of HIs for black‐capped compared to mountain chickadees suggests unidirectional backcrossing of hybrids with black‐capped chickadees in this system, or that backcrosses to mountain chickadees are less likely to be viable and fertile. Given the previously discussed influence of dominance on chickadee mate choice, and the results from Grava et al. (2012), it seems most parsimonious that hybrids are more likely to backcross with black‐capped chickadees. Higher resolution data (i.e., whole genome sequence data) are needed to establish the directionality of backcrossing and how that might influence admixture for both species.

Previous work in this system suggests that hybridization is unidirectional (Grava et al., 2012) and that a chickadee's mitochondrial DNA (mtDNA; inherited from the maternal line) always matches phenotype (Adams & Burg, 2015; Graham et al., 2021; Grava et al., 2012). Hybrid chickadees that successfully reproduce might be exclusively male due to Haldane's Rule. If hybridization is unidirectional and hybrids are produced exclusively in mountain chickadee nests, then hybrid males and females should have mountain chickadee mtDNA. If hybrid females survive and reproduce with black‐capped chickadees, mountain chickadee mtDNA should make its way into individuals with black‐capped chickadee plumage. The absence of mountain chickadee mtDNA in phenotypically black‐capped chickadee individuals suggests that female hybrids are likely sterile, which matches expectations from Haldane's Rule: hybrids of the heterogametic sex (in birds, females) are more likely to be absent, rare, or sterile relative to the homogametic sex. Importantly, the number of detected nestlings in Grava et al. (2012) was significantly higher than the number of wintering birds with intermediate HI, suggesting poor recruitment of hybrid juveniles into the adult population. Despite this, enough hybrids appear to survive to generate individuals with HI indicative of backcrossing and there is likely differential survival of hybrids based on their genetic composition. Alternatively, black‐capped chickadee plumage might be dominant to some extent, but this would not explain the absence of mountain chickadee mtDNA in hybrids with black‐capped chickadee plumage. Additional work is needed to understand the genetic basis of plumage variation and low hybrid fitness in this system.

Numerous studies have assessed species interactions using sympatry and allopatry as proxies for levels of interactions, particularly when characterizing social environments is logistically challenging (e.g., when species are distributed across large spatial areas; Brown & Wilson, 1956), but the inclusion of genetic data are rarer, especially across a broad sampling area. Our analyses revealed that, independent of factors that are well‐established proxies of social dominance status in this system (e.g., sex and age), all individuals are in poorer condition when they co‐occur with an ecologically similar relative. In the absence of sufficient genetic data, we conclude that there is a cost when black‐capped and mountain chickadees co‐occur; this may manifest itself as reduced body condition of cryptic hybrids driving the overall pattern of reduced body condition for birds in sympatry. Genetic incompatibilities in chickadee hybrids are likely given that black‐capped and mountain chickadees are not sister taxa (Harris et al., 2014), and these intrinsic incompatibilities could reasonably translate to lower body condition for hybrids. Because we do not have sufficient genetic data to address this hypothesis, we present and discuss two additional nonmutually exclusive mechanisms for low fitness in sympatry that may also be occurring when black‐capped and mountain chickadees are sympatric, independent of the cost of hybridization: interspecific competition in sympatry and range edge effects.

Reduced body condition in sympatry for both species of chickadees could be caused by direct competitive interactions over food resources. While previous studies have found microhabitat partitioning between nesting sites (Hill & Lein, 1988), the amount of overlap in diet between both species is less clear but likely occurs given that both species regularly visit seed feeders together. Broadly, direct interactions between heterospecifics are often aggressive interactions and/or physical contests over access to limited resources, such as food and space (Peiman & Robinson, 2010). Avian heterospecific contests are most common between closely related and ecologically similar species (Dhondt, 2012), and are predicted to increase as the degree of resource overlap increases (Peiman & Robinson, 2010). Reduced body condition for all individuals in sympatry suggests that competitive heterospecific interactions might decrease body condition for all individuals in areas of overlap. Peiman and Robinson (2010) conducted a meta‐analysis on heterospecific and conspecific aggressive interactions from experimental behavioral presentations using mounts and song playbacks and found that when resource overlap occurred, heterospecific aggression was higher in sympatry than in allopatry. Given the high degree of overlap between black‐capped and mountain chickadees for food resources (e.g., *Pinus* seeds), our results of lower body condition in sympatry compared to allopatry for both species support Peiman and Robinson’s (2010) meta‐analysis. Additionally, a field‐based removal experiment of two species of competing wood warblers, orange‐crowned (*Vermivora celata*) and Virginia's (*V. virginiae*) warblers, demonstrated reduced predation rates, increased adult survival rates, and increased provisioning rates for both species when the corresponding heterospecific individuals were removed. This highlights the cost of heterospecific competitive interactions for nesting sites and food resources in some passerine birds (Martin & Martin, 2001).

Another factor that could cause reduced body condition in sympatric black‐capped and mountain chickadees, independent of the cost of hybridization, is range‐edge effects: black‐capped and mountain chickadees generally co‐occur along their range limits. Range limits occur where populations are no longer self‐sustaining (Holt, 2003) and fitness tends to decrease toward range edges due to the declining availability of optimal habitat (Sexton et al., 2009). Range edges are often characterized by (1) reduced breeding opportunities as the density of conspecifics declines (Bridle & Vines, 2007), and (2) competition with adjoining competitors (Hargreaves et al., 2014). Thus, both species of chickadee may suffer reduced body condition from inhabiting suboptimal habitat at their range edges, rather than from direct interspecific competition.

Despite well‐documented habitat partitioning, both species still have an extensive range overlap in transitional habitat. Given our sampling schematic and comparative study design, we cannot discriminate between whether reduced chickadee body condition in sympatry is due to cryptic hybridization, competitive interactions with ecologically similar species, or a consequence of occurring along a range edge for either species. It is interesting, however, that *elevation* was a significant predictor of black‐capped chickadee, body condition (known‐sex and full datasets), but not mountain chickadee, where black‐capped chickadee condition decreased with increasing elevation. We did not find that mountain chickadee condition decreased with decreasing elevation, as would have been predicted if range edges alone impacted condition. This suggests that potentially both competitive interactions and range edge effects influence black‐capped chickadee body condition. Alternatively, cryptic hybrids that have black‐capped chickadee phenotypes and are produced at higher elevations might be in worse condition and be driving the pattern of reduced black‐capped chickadee condition with increasing elevation (but not the inverse of reduced mountain chickadee condition at lower elevations). This would support our conclusion that cryptic hybrid individuals in the larger dataset, none of which were identified as hybrids based on phenotype, are potentially driving the pattern of reduced body condition in sympatry for both species. Of course, intrinsic genetic hybrid incompatibilities, competitive interactions, and range edge effects are not mutually exclusive mechanisms and it is possible that multiple mechanisms are at play in this system (Hargreaves et al., 2020). Regardless of the underlying mechanism, the pattern of reduced body condition in areas of sympatry for all individuals might have important fitness consequences, and influence evolutionary trajectories, especially if reduced body condition also influences hybridization in this system.

Climate change is causing skyward contractions of high‐elevation habitats and species (Freeman et al., 2018) and skyward shifts of black‐capped chickadees could increase contact between mountain and black‐capped chickadees. This type of displacement has already been documented between black‐capped and Carolina chickadees in the Appalachians, where dominant Carolina chickadees have displaced subordinate black‐capped chickadees on multiple mountains (McQuillan & Rice, 2015). Given that hybridization appears to be unidirectional via extra‐pair copulations with male black‐capped chickadees, we expect mountain chickadees to suffer a higher cost from hybridization. We predict population cost incurred by mountain chickadees because a greater proportion of nestlings in mountain chickadee nests would presumably be lower condition hybrids. In tandem with shrinking higher elevation habitats due to climate change, this reproductive loss could place mountain chickadee populations at risk. Future studies should compare body condition metrics for mountain and black‐capped chickadees in sympatry at range edges versus range centers (e.g., control for competitive interactions) and further explore the evolutionary consequences of hybridization in this system (i.e., measure hybrid fitness) to clarify the impacts of species interactions in sympatry for these closely related songbirds.

## 5 CONCLUSIONS

We sought to characterize body condition, as a proxy for fitness, across a substantial portion of the ranges of two closely related and ecologically similar songbird species that occasionally hybridize. Overall, we found that birds in sympatry, regardless of sex or age, had significantly lower SMIs than birds in allopatry. This pattern may be driven by the inclusion of cryptic hybrids, which are likely in poor condition, in our dataset, but we do not have sufficient genetic data to test this hypothesis. Importantly, our results highlight that plumage is not a reliable indicator of hybrid status for black‐capped or mountain chickadees and support the inclusion of genetic data in future large‐scale studies that examine the costs of sympatry. Determining the causes of reduced body condition in sympatric chickadees (e.g., intrinsic genetic incompatibilities in cryptic hybrids, competitive interactions between congeners, and/or suboptimal range edge habitat) is an exciting avenue of future research. Continued rapid climate change may allow black‐capped chickadee ranges to expand and will potentially increase range overlap between mountain and black‐capped chickadees. Quantifying the impacts of sympatry on individual fitness, and overall population viability, has implications for understanding how individual‐level traits (i.e., hybrid ancestry and body condition) impact population‐level dynamics such as species distributions. The mechanisms driving sporadic hybridization between black‐capped and mountain chickadees also remain largely unexplored and are an avenue of future research.

## CONFLICT OF INTEREST

The authors of this paper have no conflicts of interest to declare.

## AUTHOR CONTRIBUTION

**Kathryn C Grabenstein:** Conceptualization (lead); Formal analysis (lead); Funding acquisition (equal); Methodology (equal); Visualization (lead); Writing – original draft (lead); Writing – review & editing (equal). **Ken A Otter:** Data curation (supporting); Funding acquisition (equal); Methodology (equal); Project administration (equal); Resources (equal); Writing – review & editing (equal). **Theresa Burg:** Data curation (equal); Funding acquisition (equal); Methodology (equal); Resources (equal); Writing – review & editing (equal). **Scott A. Taylor:** Conceptualization (supporting); Formal analysis (supporting); Funding acquisition (equal); Methodology (equal); Resources (equal); Supervision (lead); Writing – original draft (supporting); Writing – review & editing (equal).

## Supporting information

Fig S1

Fig S2

## Data Availability

Data are publicly available at Data Dryad DOI https://doi.org/10.5061/dryad.qrfj6q5j7
