# Supplementary figures and images for: Sympatry leads to reduced body condition in chickadees that occasionally hybridize
Source: Ecol Evol. 2022 Apr 1;12(4):e8756. doi: 10.1002/ece3.8756 (PMC8975787; doi:10.1002/ece3.8756)

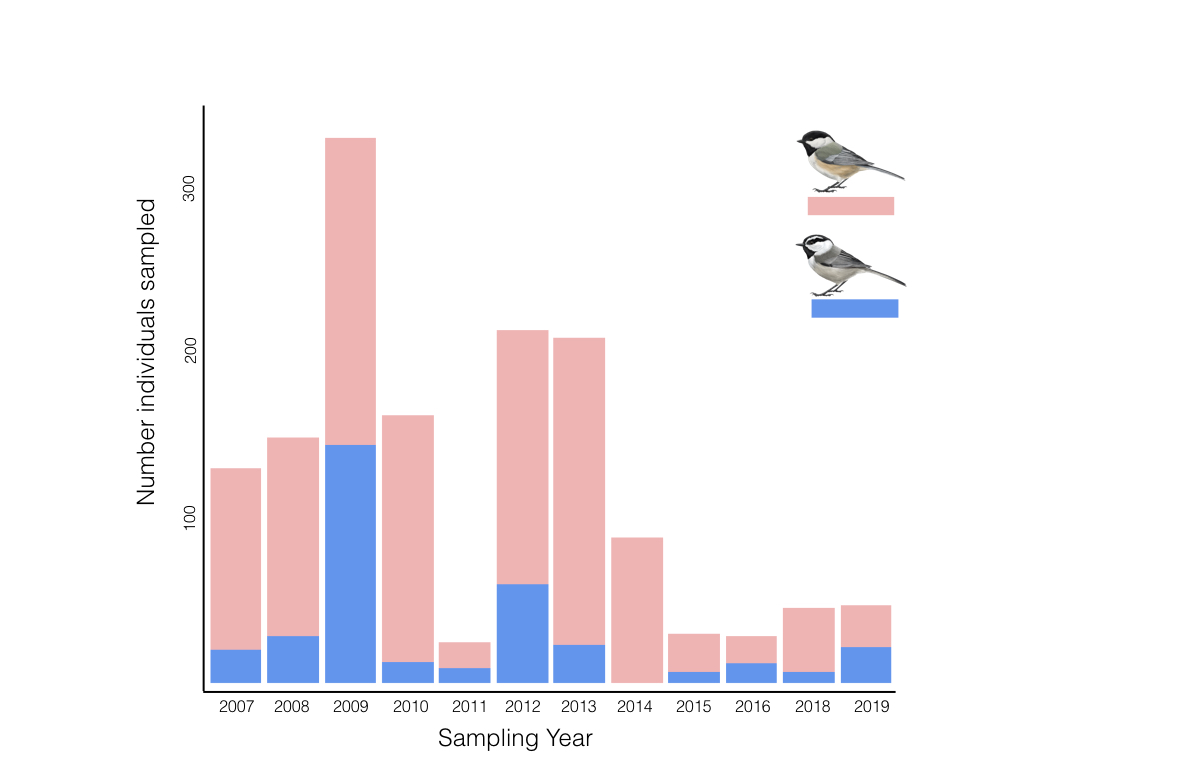

Supplement: Supplementary file 1 — Fig S1 [file ECE3-12-e8756-s001.jpeg]

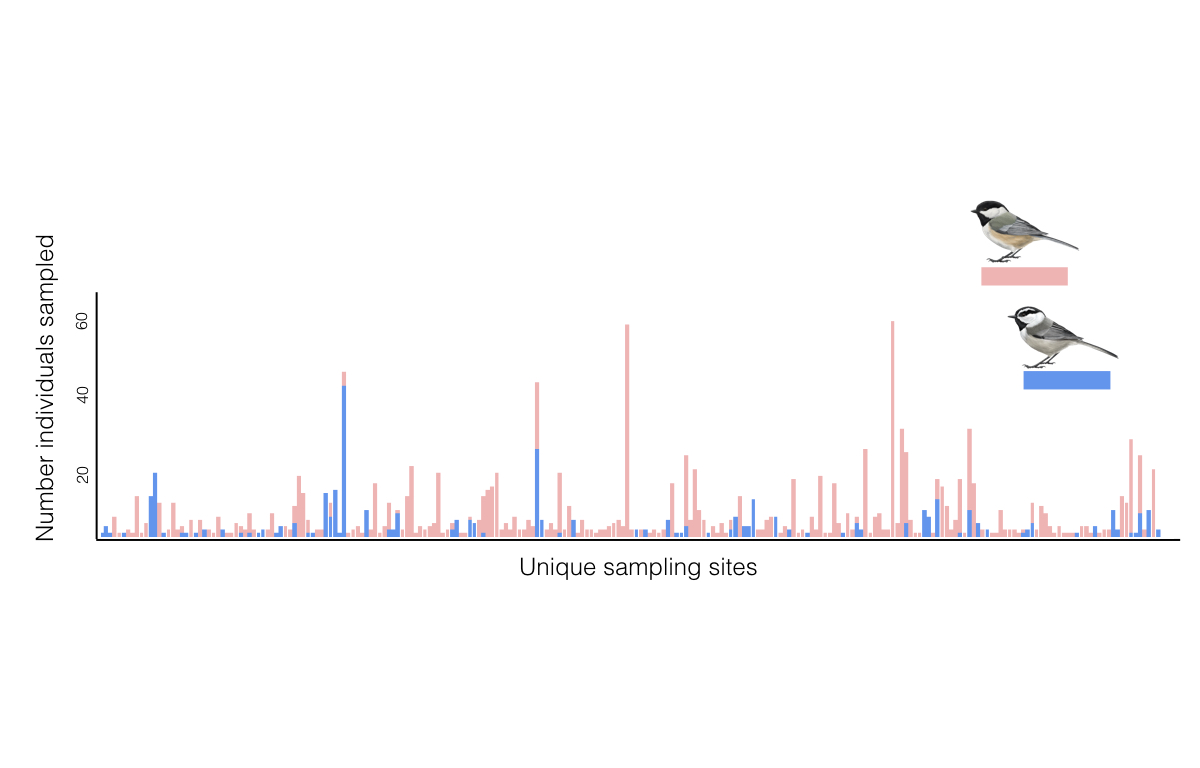

Supplement: Supplementary file 2 — Fig S2 [file ECE3-12-e8756-s002.jpeg]
